# The multifaceted role of RNA-based regulation in plant stress memory

**DOI:** 10.3389/fpls.2024.1387575

**Published:** 2024-04-26

**Authors:** Wei-Bo Xu, Fan Cao, Peng Liu, Kang Yan, Qian-Huan Guo

**Affiliations:** ^1^ State Key Laboratory of Crop Biology, College of Life Sciences, Shandong Agricultural University, Taian, Shandong, China; ^2^ Donald Danforth Plant Science Center, St. Louis, MO, United States

**Keywords:** stress memory, environmental stresses, microRNA, small interfering RNA, long noncoding RNA, alternative splicing

## Abstract

Plants have evolved interconnected regulatory pathways which enable them to respond and adapt to their environments. In plants, stress memory enhances stress tolerance through the molecular retention of prior stressful experiences, fostering rapid and robust responses to subsequent challenges. Mounting evidence suggests a close link between the formation of stress memories and effective future stress responses. However, the mechanism by which environmental stressors trigger stress memory formation is poorly understood. Here, we review the current state of knowledge regarding the RNA-based regulation on stress memory formation in plants and discuss research challenges and future directions. Specifically, we focus on the involvement of microRNAs (miRNAs), small interfering RNAs (siRNAs), long non-coding RNAs (lncRNAs), and alternative splicing (AS) in stress memory formation. miRNAs regulate target genes via post-transcriptional silencing, while siRNAs trigger stress memory formation through RNA-directed DNA methylation (RdDM). lncRNAs guide protein complexes for epigenetic regulation, and AS of pre-mRNAs is crucial to plant stress memory. Unraveling the mechanisms underpinning RNA-mediated stress memory formation not only advances our knowledge of plant biology but also aids in the development of improved stress tolerance in crops, enhancing crop performance and global food security.

## Introduction

As sessile organisms, plants must respond and adapt to fluctuating, and sometimes near-lethal, environmental conditions over their entire lifespan (Trivedi et al., 2020; Zhang et al., 2022). To endure adverse environmental conditions, plants have evolved stress tolerance mechanisms, including the establishment of a “stress memory” (Crisp et al., 2016; Hilker and Schmülling, 2019; Liu et al., 2022). Stress memory is formed through transient exposure to mild or severe stress and allows primed plants to respond robustly and swiftly to subsequent stressors, facilitating their recovery (Crisp et al., 2016; Liu et al., 2022). Understanding how plants stress memory works is crucial for improving crop resilience and productivity, which can help ensure food security in the face of changing environmental conditions and growing global food demands.

Following the initial stress exposure, stress memory modulates the expression of key genes through epigenetic modifications such as chromatin re-modelling, DNA methylation, nucleosome positioning, histone modification, and non-coding RNA-mediated regulation (Liu et al., 2022). In addition, a growing body of evidence suggests that stress memory involves RNA-mediated regulation via gene silencing and/or activation (Crisp et al., 2016). Here, we review the roles played by diverse RNAs in plant stress memory by summarizing recent research advances and providing generalized examples. MicroRNAs (miRNAs) and small interfering RNAs (siRNAs) are involved in plant stress memory via post-transcriptional gene silencing and RNA-directed DNA methylation, respectively (Song et al., 2019; Li et al., 2020). Long non-coding RNAs (lncRNAs) and alternative splicing (AS) also play crucial roles in stress memory (Ohama et al., 2017; Chaudhary et al., 2019; Yu et al., 2019; Ling et al., 2021). Finally, we investigate the mechanisms of RNA-mediated stress memory in plants and suggest possible future research directions (**Table 1**).

**Table 1:** A summary of RNA-based Regulation on Plant Stress Memory.

Gene	Specie	Stress responses	Type	Function	References
miR156	*Arabidopsis*	Heat	miRNA	miR156-SPL module mediates the response to recurring heat stress.	(Cho et al., 2012; Stief et al., 2014a, Stief et al., 2014b)
miR824	*Arabidopsis, Brassicaceae*	Heat	miRNA	miR824/AGAMOUS-LIKE16 module integrates recurring heat stress.	(Szaker et al., 2019)
miR168	*Brassica*	Heat	miRNA	Altered expression of miR168 in parental *B. rapa* plants exposed to heat stress and in the untreated progeny.	(Bilichak et al., 2015)
Tae-miR531_L-2	*Wheat*	Drought	miRNA	Overexpression of the tae-miR531_L-2 improves the drought tolerance.	(Yue et al., 2022)
Osa-miR168a-3p_L-3, ata-miR169-3p	*wheat*	Water-Deficit, Heat	miRNA	transgenerational effects of water-deficit and heat stress in the same genotypes.	(Liu et al., 2020)
miR398, miR408	*coffee*	Drought	miRNA	miR398 and miR408 were up-regulated by the drought cycles in coffee.	(Guedes et al., 2018)
Ttu-miR160	*Wheat*	Water-deficit	miRNA	Small RNAs and their targets are associated with the transgenerational effects of water-deficit stress.	(Liu et al., 2021)
siR255, siR1511	*Arabidopsis*	Heat	siRNA	SGIP1-mediated SGS3 degradation leads to inhibited biosynthesis of trans-acting siRNA.	(Liu et al., 2019)
*ONSEN-specific siRNAs*	*Arabidopsis*	Heat	siRNA	siRNA-related pathway mediated *ONSEN* transcriptional activation and ONSEN transposition serves as a transgenerational form of heat stress memory.	(Ito et al., 2011; Matsunaga et al., 2012, Matsunaga et al., 2015)
TCONS_00028567	*Rice*	Drought	IncRNA	IncRNA participate in rice short-term drought memory.	(Li et al., 2019)
XLOC_033252	*Switchgrass*	Dehydration	IncRNA	The levels of IncRNAs increased in both the first and second drought cycles.	(Zhang et al., 2018)
*COOLAIR*	*Arabidopsis*	Cold	IncRNA	*COOLAIR* in the coordinated switching of chromatin states that occurs during cold, linking transcriptional shutdown with epigenetic silencing.	(Csorba et al., 2014: Nielsen et al., 2024)
*COLDAIR*	*Arabidopsis*	Cold	IncRNA	*COLDAIR* is required for establishing stable repressive chromatin at FLC through its interaction with PRC2.	(Heo and Sung, 2011; Kim and Sung, 2017; Kim and Sung, 2017)
*COLDWRAP*	*Arabidopsis*	Cold	IncRNA	*COLDWRAP* is derived from the repressed promoter of FLC and is necessary for the establishment of the stable repressed state of FLC by vernalization.	(Kim and Sung, 2017; Kim and Sung, 2017)
*RSZ22, RZIA*	*Pinus*	Heat	Alternative Splicing	Stress-responsive AS events participate in the establishment of long-term thermos- memory	(Roces et al., 2022)
*HSFBI, HSFB2a, HSFA2*	*Arabidopsis*	Heat	Alternative splicing	AS may contribute to heat stress-induced memory	(Ling et al., 2018; Sanyal et al., 2018)

## The role of miRNAs in plant stress memory: post-transcriptional silencing

**Figure 1:**
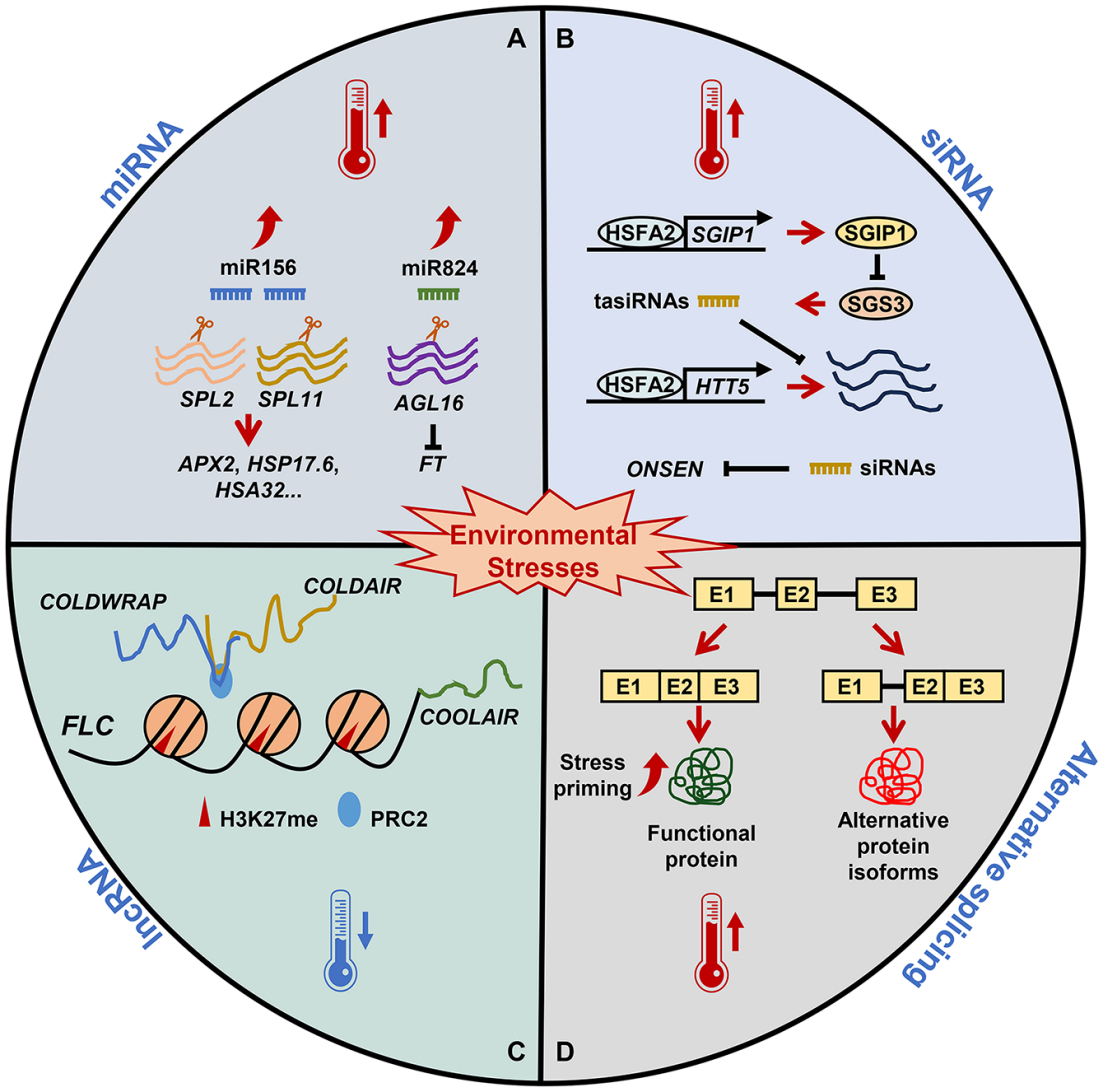
The role of RNA-based regulation in plant stress memory. Under nonstress conditions, miRNAs, siRNAs, lncRNAs, and alternative splicing are essential for plant growth and development. **(A)** Under heat stress, miR156 expression is induced and *SPL* genes are post-transcriptionally downregulated, affecting the expression of heat stress memory-related genes. Parallel to miR824 induction, its target *AGL16* is decreased. *AGL16* downregulation in response to heat leads to a fine-tuning of FT. **(B)** Heat stress upregulates *HSFA2*, which activates SGIP1 to trigger the transgenerational degradation of SGS3, leading to the suppression of tasiRNA biosynthesis. The reduced tasiRNA levels converge to activate *HTT5*. In addition, siRNA-mediated regulation and the initiation of transgenerational transposition of *ONSEN* are involved in heat stress memory. **(C)** Winter cold triggers high levels of H3K27me3 at FLC mediated by the PRC2 complex. lncRNAs, including *COOLAIR*, *COLDAIR*, and *COLDWRAP*, are important for *FLC* repression. *COOLAIR* decreases H3K36me3 at FLC; *COLDAIR* recruits PRC2 and promotes *FLC* repression; and *COLDWRAP* may help PRC2 spread to the *FLC* promoter and stabilize H3K27me3. **(D)** Exposure to sub-lethal heat stress results in the priming of plants, which establishes splicing-linked heat-stress memory. This heat priming executes correct splicing and ensures the functional RNA/proteins resulting in an effective adaptive response that ensures the survival of plants.

In plants, miRNAs regulate gene expression and/or silencing by binding to complementary sequences within target messenger RNAs (mRNAs), resulting in translational repression and/or transcript degradation (Sunkar et al., 2012; Ha and Kim, 2014). miRNAs are critical for regulating plant growth and reproduction, as well as biotic and abiotic stress responses (Wang et al., 2009; Zhao et al., 2019; Liebsch and Palatnik, 2020; Li et al., 2023; Xu et al., 2023). Recent research suggests that miRNAs may also be key regulators of plant stress memory. Specifically, miRNAs can respond quickly to environmental and developmental cues via the post-transcriptional silencing of stress-responsive target genes (**Figure 1A**).

Most recently, improved high-throughput sequencing techniques have revealed the close relationship between miRNAs and stress memory. With drought pretreatment, 195 miRNAs, including 186 drought memory-specific and nine significantly differentially expressed shared miRNAs, were identified as candidate drought memory-related miRNAs in wheat (*Triticum aestivum*) (Yue et al., 2022). In *Arabidopsis thaliana*, overexpression of the wheat drought memory-related miRNA tae-miR531_L-2 significantly improves drought tolerance in transgenic plants (Yue et al., 2022). A recent study of the miRNAome of wheat seedlings subjected to water deficit and heat stress revealed the long-term impact of stress on plant physiology and gene regulation and suggested that miRNAs and their target genes play important roles in transgenerational stress adaptation (Liu et al., 2020). In coffee (*Coffea arabica*) subjected to repeated cycles of drought, the transcriptional levels of miRNA-guide stress-related genes are different, which exhibit distinct transcriptional memory behavior (Guedes et al., 2018). Specifically, their complex regulation of target *V-myb myeloblastosis viral oncogene* (*MYB*) homologs highlights the crucial role of MYB at the crossroads of plant miRNA-mediated stress memory (Guedes et al., 2018).

Recent work has also revealed that multiple miRNAs participate in heat stress memory (Stief et al., 2014b). Mutant plants with impaired small RNA (sRNA) biogenesis, specifically *ago1* (*Argonaute1*) and *dcl1* (*Dicer-like1*) mutants, are unable to acquire thermotolerance, indicating that heat stress memory requires miRNA intermediation (Stief et al., 2014a). Isoforms of *miR156* are highly-induced following exposure to heat stress, and repression of their target genes *SQUAMOSA promoter binding protein-like 2* (*SPL2*) and *SPL11*, which is required for the transcriptional induction of heat stress memory genes, including *ASCORBATE PEROXIDASE 2* (*APX2*), *HEAT STRESS ASSOCIATED 32* (*HSA32*), *HEAT SHOCK TRANSCRIPTION FACTOR A2* (*HSFA2*), *HEAT SHOCK PROTEIN 17.6A* (*HSP17.6A*) and *HEAT SHOCK PROTEIN 22* (*HSP22*) (Stief et al., 2014a). Moreover, *miR156h* overexpression-induced acquired thermotolerance results from the increased expression of heat stress memory-related genes (Stief et al., 2014a). Notably, heat stress-induced *miR156* induction has been observed in alfalfa (*Medicago sativa*), field mustard (*Brassica rapa*), ginkgo (*Ginkgo biloba*), banana (*Musa acuminata*), safflower (*Carthamus tinctorius*), and wheat (*T. aestivum*), suggesting that this miRNA may have conserved functions in both development and heat stress memory (Wang et al., 2014; Ragupathy et al., 2016; Matthews et al., 2019; Zhu et al., 2019; Chang et al., 2020; Kouhi et al., 2020). As miR156 is an important regulator of developmental transitions, this signaling module may be used to integrate stress memory and plant development (Cho et al., 2012; Cheng et al., 2021).

Similarly, heat-responsive induction of *miR824* appears to be dependent on *HEAT* SHOCK TRANSCRIPTION FACTOR A1 (HSFA1) in *A. thaliana* (Szaker et al., 2019). HSFA1a directly regulating miR824 promoter to activate the transcription under heat stress, and *AGAMOUS LIKE 16* (*AGL16*) is a target gene of miR824. *miR824*-dependent *AGL16* downregulation is primarily manifested post stress exposure, suggesting that *miR824* may post-transcriptionally modulate *FLOWERING LOCUS T* (*FT*)-driven development in response to environmental signals (Hu et al., 2014; Szaker et al., 2019). Notably, heat-mediated regulation of the *miR824*/*AGL16* module is conserved in multiple Brassicaceae species (Kutter et al., 2007). This module integrates multiple abiotic stimuli under complex climatic conditions, and therefore may hold considerable potential for enhancing plant stress resistance (Kutter et al., 2007). In *B. rapa*, the differential expression of *bra-miR168* following heat stress is correlated with *braAGO1* transcription, suggestive of their potential roles as key regulators of transgenerational stress memory (Bilichak et al., 2015).

Overall, the involvement of post-transcriptional regulatory miRNA modules in plant stress memory highlights the flexibility of plants to effectively respond and adapt to fluctuating and stressful environmental conditions. Additionally, as an indicator of their long-term adaptability, stress memory allows plants to respond rapidly and robustly to future stressors, effectively acting as an inoculation.

## The role of siRNAs in stress memory: RNA-directed DNA methylation

siRNAs are a class of non-coding RNA molecules produced through the processing of long double-stranded RNA (dsRNA) precursors (Lee et al., 2023). Similarly to miRNAs, siRNAs actively participate in RNA interference (RNAi) by binding to complementary mRNA sequences, resulting in mRNA cleavage and subsequent degradation (Liu et al., 2023). Notably, siRNAs can also induce silencing and RNA-directed DNA methylation (RdDM) to enforce epigenetic states, which may be involved in plant stress memory (Liu et al., 2019).

In *A. thaliana*, heat stress-induced *HSFA2* expression results in the suppression of the SUPPRESSOR OF GENE SILENCING 3 (SGS3) protein, which is involved in siRNA production (Liu et al., 2019) (**Figure 1B**). This pathway leads to inhibited siRNA biosynthesis, a decrease in methylation, and the suppression of transposons and stress-responsive loci, thus allowing stress memory to persist (Liu et al., 2019). Similarly, a retrotransposon known as *ONSEN* (Japanese for “hot spring”) is significantly activated in response to heat stress because it is targeted by HSFA1 and HSFA2 (Ito et al., 2011; Matsunaga et al., 2012). Heat-induced *ONSEN* transcription and transposition are promoted in mutant plants with impaired siRNA biogenesis (Matsunaga et al., 2012). Although both the *ONSEN* transcripts and extrachromosomal DNA decayed over time, new *ONSEN* insertions were observed in the progeny of stressed siRNA-deficient plants (Matsunaga et al., 2015). An siRNA-mediated mechanism is involved in the new insertions happen in the progeny. Heat activated *ONSEN* was transposed to the next generation and increased in copy number in the host genome (Matsunaga et al., 2015). Together, these studies highlight the involvement of siRNA-guide epigenetic mechanisms in the formation of transgenerational stress memory.

## Emerging roles of long non-coding RNAs in stress memory

lncRNAs, which are typically longer than 200 nucleotides (nt), are a large and diverse class of eukaryotic genes which contribute to an array of regulatory processes (Palos et al., 2023). For example, lncRNAs play an important role in coping with environmental stress during plant growth and development (Song et al., 2021). Specifically, lncRNAs mediate epigenetic modifications, and by studying their participation in stress memory we may begin to unravel the intricate interplay between non-coding RNAs and epigenetic regulation (**Figure 1C**).

Strand-specific whole-transcriptome RNA sequencing of repeatedly drought-stressed rice (*Oryza sativa*) revealed that the lncRNA *TCONS_00028567*, a predicted precursor of the miRNA are strongly upregulated at the second drought treatment stage, but downregulated at the three re-water treatment stage (Li et al., 2019). Such expression variability of *TCONS_00028567* is related to the repeatedly short-term drought treatment, suggesting that *TCONS_00028567* may regulate short-term drought memory in rice. In switchgrass (*Panicum virgatum*), lncRNAs targeting the biosynthesis of ABA and trehalose were upregulated during the first and second drought cycles, but lncRNAs regulating ethylene signaling were suppressed in the second drought cycle, thereby preventing leaf senescence and supporting plant development under stressful conditions (Zhang et al., 2018).

In *A. thaliana*, low-temperature-responsive epigenetic modifications are induced during vernalization, resulting in the repression of the *FLOWERING LOCUS C* (*FLC*) gene until flowering commences (Zhu et al., 2021). The *FLC* locus includes three different cold-induced lncRNAs: *COOLAIR*, *COLDAIR*, and *COLDWARP* (Hung et al., 2022). *COOLAIR* is highly induced when plants experience a dip below freezing temperature, likely analogous to the first frost in autumn. Then, upregulation of *COOLAIR* leads to decreased *FLC* expression and disruption of the synchronized replacement of H3K36 with H3K27me3 methylation at the *FLC* nucleation site (Csorba et al., 2014; Nielsen et al., 2024). Both *COLDAIR* and *COLDWRAP* bind to *FLC* chromatin, and thus stably silence *FLC* by recruiting PHD-PRC2 to its specific chromatin location in response to cold temperatures (Heo and Sung, 2011; Kim and Sung, 2017). Thus, the cold-induced Polycomb nucleation mechanism locks in the FLC silenced transcriptional state to maintain the epigenetic memory of cold exposure (Zhao et al., 2021). It appears that lncRNAs act as guides for protein complexes mediating epigenetic regulation, facilitating the formation of cold stress memory in plants.

Overall, these findings highlight the importance of lncRNAs to the formation of stress memory in plants. We anticipate that an increasing number of lncRNAs will be discovered across a diverse range of plant species, and that many of these will be found to be involved in plant stress memory formation and enhanced stress tolerance.

## The relationship between AS and stress memory

AS results in the generation of multiple transcripts from the same gene, thereby increasing proteomic diversity and regulating gene expression and mRNA levels (Chaudhary et al., 2019). To maximize metabolic efficiency under stressful conditions, plants may make more proteins with disordered domains via AS in order to diversify substrate specificity and maintain sufficient regulatory capacity (Ling et al., 2021). According to recent research, splicing-linked memory formation during the priming phase is crucial for guaranteeing the availability of correctly-spliced transcripts or proteins which are essential for increased stress tolerance (**Figure 1D**).

Recent studies in *A. thaliana* suggest that AS may be a novel component of heat shock memory formation (Ling et al., 2021). For example, priming plants with non-lethal heat stress results in the de-repression of splicing following a second exposure to heat stress, while non-primed plants exhibit significant splicing repression. An array of heat shock protein (HSP) genes, such as *HSP21*, *HSP101*, *HSP70.10*, *HSP70.6*, *HSP90.5*, and *HSP100.3*, have been found to undergo AS in response to heat stress/priming, primarily through intron retention (Ling et al., 2018). Specifically, the levels of intron-retained isoforms were found to be higher during heat shock, with the exception of *HSP70.17*. The constitutively-spliced isoform of *HSP70.10* was mainly expressed during heat priming, although multiple isoforms were observed during other phases (Ling et al., 2018). In contrast, several isoforms of *HSP90.6* were observed during the priming phase, but not during other phases (Ling et al., 2018).

Such observations link “splicing memory” to the ability of plants to survive subsequent, and perhaps otherwise lethal, heat stress events. Therefore, priming-induced splicing memory may represent a general feature of the plant heat stress response (Sanyal et al., 2018). AS-mediated stress memory formation may itself be mediated by epigenetic coding. Integrated studies in pine (*Pinus* spp.) implicate AS as an important mechanism mediating stress response and memory. Specifically, changes in spliceosome-related proteins were observed during heat stress and recovery, as in the cases of *SERINE/ARGININE-RICH SPLICING FACTOR RSZ22* (*RSZ22*), *GLYCINE-RICH RNA-BINDING PROTEIN RZ1A* (*RZ1A*), and *UNCHARACTERIZED PROTEIN DUF4050* (*DUF4050*) (Roces et al., 2022). Moreover, exon-skipping events may be induced during drought memory formation in rice (*O. sativa*), with 920 drought memory-associated genes exhibiting differential AS patterns (Yang et al., 2022).

Plants undergo changes in their gene expression patterns in response to stress exposure. AS contributes to both protein diversity and functional plasticity, allowing plants to adapt to adverse conditions (Chaudhary et al., 2019). For example, certain splice variants may be preferentially produced in response to specific stressors, leading to the activation of tailored defense mechanisms (Kutter et al., 2007). This process allows plants to retain a “memory” of previous stress events, enabling them to mount faster and more effective responses upon subsequent exposure to similar stressors. By deciphering the intricate connections between AS and stress response, researchers may be able to develop crop varieties with enhanced resilience to environmental stressors.

## Conclusion and future prospects

In this review, we summarized the role of non-coding RNAs such as miRNAs, siRNAs, and lncRNAs, as well as AS, in the regulation of plant stress memory formation. We highlighted the interconnected regulatory pathways which enable plants to remember past stress events and to use those stored responses to better adapt to new challenges. The involvement of non-coding RNAs in stress memory is demonstrated through their ability to quickly respond to environmental and developmental cues, enhancing stress tolerance and contributing to epigenetic regulation.

However, many questions remain regarding RNA-mediated stress memory formation. First, how do different stressors differentially mediate the formation of stress memories in plants? Second, how can acquired stress memories be efficiently transferred to offspring to enhance the stress resistance of subsequent generations? Third, are there alternative mechanisms (i.e., other than epigenetic) of stress memory transmission between generations? Answering these questions will significantly improve our understand of RNA-mediated stress memory formation, thereby allowing the development of improved crop varieties with enhanced stress tolerance. This research will be crucial in addressing global food security challenges posed by climate change, population growth, and other factors. By unraveling the mysteries of RNA-mediated stress memory formation, we can not only develop more resilient crop varieties but also lay the foundation for sustainable agriculture practices that address the complex challenges of climate change on a global scale.

## Author contributions

W-BX: Writing – original draft, Writing – review & editing. FC: Writing – review & editing. PL: Writing – review & editing. KY: Writing – original draft, Writing – review & editing. Q-HG: Writing – original draft, Writing – review & editing.
